# Laboratory-Acquired *Brucella abortus* Infection Mimicking Autoimmune Disease: A Case Report with Genomic Confirmation

**DOI:** 10.3390/pathogens15050460

**Published:** 2026-04-23

**Authors:** Judit Henczkó, Zsuzsa Kienle, János Kádár, Enikő Kádár-Hürkecz, Katalin Tárnoki-Boross, Panna Sütő, Bernadett Pályi, Ákos Tóth, Katalin Kamotsay, Zsuzsanna Molnár, Zoltan Kis

**Affiliations:** 1 Department of National Biosafety Laboratory, National Center for Public Health and Pharmacy, 1097 Budapest, Hungary; 2 Doctoral College, Semmelweis University, 1091 Budapest, Hungary; 3 Department of Bacteriology, Parasitology and Mycology, National Center for Public Health and Pharmacy, 1097 Budapest, Hungary; 4 Department of Hematology, Central Hospital of Southern Pest—National Institute of Hematology, 1097 Budapest, Hungary; 5 Central Laboratory, Microbiology Profile, Central Hospital of Southern Pest, 1097 Budapest, Hungary; 6 Communicable Diseases and Immunization Unit, National Center for Public Health and Pharmacy, 1097 Budapest, Hungary; 7 Institute of Medical Microbiology, Faculty of Medicine, Semmelweis University, 1085 Budapest, Hungary

**Keywords:** brucellosis, laboratory-acquired infection, zoonotic disease, autoimmune mimicry, whole-genome sequencing, elevated ferritin levels, antinuclear antibody (ANA) positivity

## Abstract

Background: Brucellosis is a globally distributed zoonotic disease characterized by highly variable clinical manifestations that may mimic systemic autoimmune and inflammatory disorders. In Europe, where the incidence of brucellosis is relatively low, limited clinical awareness may contribute to delayed diagnosis and inappropriate management. In addition to zoonotic transmission, *Brucella* species are a well-recognized cause of laboratory-acquired infections (LAIs) among microbiology laboratory personnel. Methods: We report a case of laboratory-acquired *Brucella abortus* infection in a young woman presenting with undulant fever, arthralgia, systemic inflammation, elevated ferritin levels, and antinuclear antibody (ANA) positivity. Microbiological confirmation was achieved through serological testing (ELISA), repeat blood cultures, species-specific quantitative PCR, and whole-genome sequencing (WGS) followed by core genome multilocus sequence typing (cgMLST). Results: Initial laboratory evaluation revealed elevated C-reactive protein, mildly increased ferritin levels (146 ng/mL), abnormal liver enzyme levels, and rising ANA titers (from 1:160 to 1:320), raising suspicion of a systemic autoimmune disorder and prompting consideration of corticosteroid therapy. Although the initial blood culture was negative, subsequent molecular diagnostics and repeat cultures confirmed *B. abortus* infection. Epidemiological investigation suggested a possible occupational exposure in a diagnostic microbiology laboratory, consistent with a laboratory-acquired infection. Genomic analysis classified the isolate as sequence type 1 (ST1) and demonstrated zero allelic differences compared with the ST1 reference strain. Targeted antimicrobial therapy resulted in complete clinical recovery, supporting an infection-triggered immune response rather than primary autoimmunity. Conclusions: Acute brucellosis should be considered in the differential diagnosis of febrile syndromes accompanied by autoimmune-like laboratory abnormalities, even in low-incidence regions. This case highlights the diagnostic challenges posed by laboratory-acquired brucellosis and underscores the importance of early microbiological investigation and strict biosafety awareness in laboratory settings.

## 1. Introduction

Brucellosis, or ‘Malta fever’ and ‘undulant fever’, is one of the most common zoonoses, causing approximately half a million new cases annually worldwide [1,2]. *Brucella melitensis* (sheep, goat), *Brucella abortus* (bovine, bison), and *Brucella suis* (hare, pig) are the most prevalent in human infections [1]. The estimated incidence in endemic areas varies from less than 0.01 to more than 200 cases per 100,000 people [3]. Despite extensive eradication efforts in animals, brucellosis remains endemic in many countries. Nevertheless, the incidence of human infections in Europe remains comparatively low. In 2022, 199 cases were documented, and a modest increase was observed in 2023, with 259 cases reported in the European Union, mostly in Greece, Portugal, France, Spain, and Germany [3,4]. Since 1985, the bovine population in Hungary has been free of *B. abortus*, although clinically relevant *B. abortus* biovar 1 and *B. suis* biovar 2 are still present in wildlife [2,5]. Autochthonous human brucellosis cases are extremely rare in Hungary; only six cases have been reported in the past 40 years, the majority of which were travel-related (National Center for Public Health and Pharmacy, Hungary, data not published). Infection in humans is primarily acquired through the consumption of contaminated food—particularly unpasteurized dairy products—direct contact with infected animals or animal tissues, inhalation of aerosolized particles, or occupational exposure. The incubation period typically ranges from 2 to 6 weeks but may extend to several months, complicating epidemiological identification [1,2]. Clinically, brucellosis is considered a “great imitator” due to its broad and nonspecific symptomatology. Patients may present with prolonged fever, fatigue, osteoarticular manifestations, neurological involvement, cardiovascular complications, or hematological abnormalities [1,2,6,7]. Brucellosis is often misdiagnosed as an autoimmune disorder, such as hemolytic anemia, autoimmune glial fibrillary acidic protein (GFAP) astro cytopathy, systemic lupus erythematosus, or other malignant diseases [6,8,9,10,11,12]. Elevated ferritin levels are frequently observed and may reflect macrophage activation and systemic inflammation [2,11]. Laboratory diagnosis relies primarily on serological testing, supported by blood culture and polymerase chain reaction assays, which are highly specific across disease stages. Whole genome sequencing currently represents the highest-resolution method for strain identification and molecular epidemiological confirmation [1,11,13,14]. In addition to natural zoonotic transmission, laboratory-acquired infections represent a well-recognized occupational hazard, particularly for microbiology laboratory personnel handling cultures or clinical specimens containing *Brucella* species; given its low infection dose, brucellosis belongs to the top five laboratory acquired infections (LAI) [15,16,17,18,19]. Here, we report a case of laboratory-acquired *Brucella abortus* infection that initially mimicked an autoimmune disease.

## 2. Detailed Case Description

A 24-year-old female patient presented in January 2025 with undulant fever and arthralgia. She initially consulted her general practitioner, where a urinary tract infection was suspected and a single-dose fosfomycin regimen was prescribed. As no clinical improvement was observed, and fever with arthralgia persisted, she was referred in February 2025 to the National Institute of Haematology and Infectology for further evaluation.

At admission, routine laboratory investigations and blood cultures were obtained, and the patient was discharged pending results. Laboratory investigations at initial presentation revealed ferritin 146 ng/mL, white blood cell count 4.82 G/L, hemoglobin 121 g/L, hematocrit 36%, platelets 268 G/L, and C-reactive protein 34 mg/L. Liver enzyme levels showed alanine aminotransferase (ALT) 147 U/L and aspartate aminotransferase (AST) 53 U/L. Complement levels were slightly elevated (C3 1.80 g/L, C4 0.50 g/L). Antinuclear antibodies (ANA) were positive by HEp-2 indirect immunofluorescence at a titer of 1:160 with a granular pattern. Serum protein electrophoresis demonstrated elevated beta-1 globulin 10.6%, beta-2 globulin 6.4%, gamma globulin 25.7%, and IgG4 1.71%. A single set of aerobic and anaerobic blood cultures (BD BACTEC system; 5 mL per bottle), collected during a febrile episode, remained negative after five days of incubation. Taken together, the combination of persistent fever, arthralgia, elevated inflammatory markers, mild hyperferritinemia, positive ANA with increasing titers, and liver enzyme abnormalities raised suspicion of a systemic autoimmune or inflammatory disorder, prompting consideration of corticosteroid therapy.

The patient was recalled for follow-up, and corticosteroid therapy was considered. In parallel, Brucella serology was requested according to institutional diagnostic protocols. A native serum sample was forwarded to the National Center for Public Health and Pharmacy, Department of Microbiological Reference Laboratories. An in-house standard agglutination test revealed a high *Brucella* antibody titer of 1:1240. Confirmation by commercial ELISA (SERION GmbH, Würzburg, Germany) demonstrated markedly elevated antibody levels (IgA > 100 U/mL, IgG > 500 U/mL, IgM 56 U/mL). Initial serum in-house quantitative PCR (qPCR) targeting the *bcsp*31 gene was negative [20]. Therefore, additional specimens, including EDTA blood and two further blood culture sets were requested. Subsequent qPCR performed from EDTA blood was positive for *bcsp*31, and species-specific IS711/*alk*B in-house qPCR confirmed *Brucella abortus* [20].

One of the repeated blood cultures (BACTEC Standard Aerobic, Becton Dickinson, Franklin Lakes, NY, USA) supplemented with brucella broth (NutriSelect-Plus, Sigma-Aldrich, Darmstadt, Germany) became positive after 72 h of incubation. Subculture on in-house 10% sheep blood agar yielded semi-transparent, smooth, round colonies after 24 h (Isolate 10073-2025). Identification by MALDI-TOF MS (Bruker Biotyper Sirius, database version 7.0) confirmed *Brucella abortus*.

Antimicrobial susceptibility testing was performed by using MIC Test Strip (Liofilchem, Roseto degli Abruzzi, Italy). While the lack of EUCAST breakpoints for *B. abortus* prevented specific interpretation of the data, the following MICs values were obtained: ceftriaxone, 0.125 μg/mL; doxycycline, 0.125 μg/mL; gentamicin, 0.5 μg/mL; ciprofloxacin, 0.06 μg/mL; levofloxacin, 0.25 μg/mL; rifampicin, 0.5 μg/mL; streptomycin, 0.06 μg/mL; and trimethoprim–sulfamethoxazole, 0.064 μg/mL, respectively.

**Figure 1:**
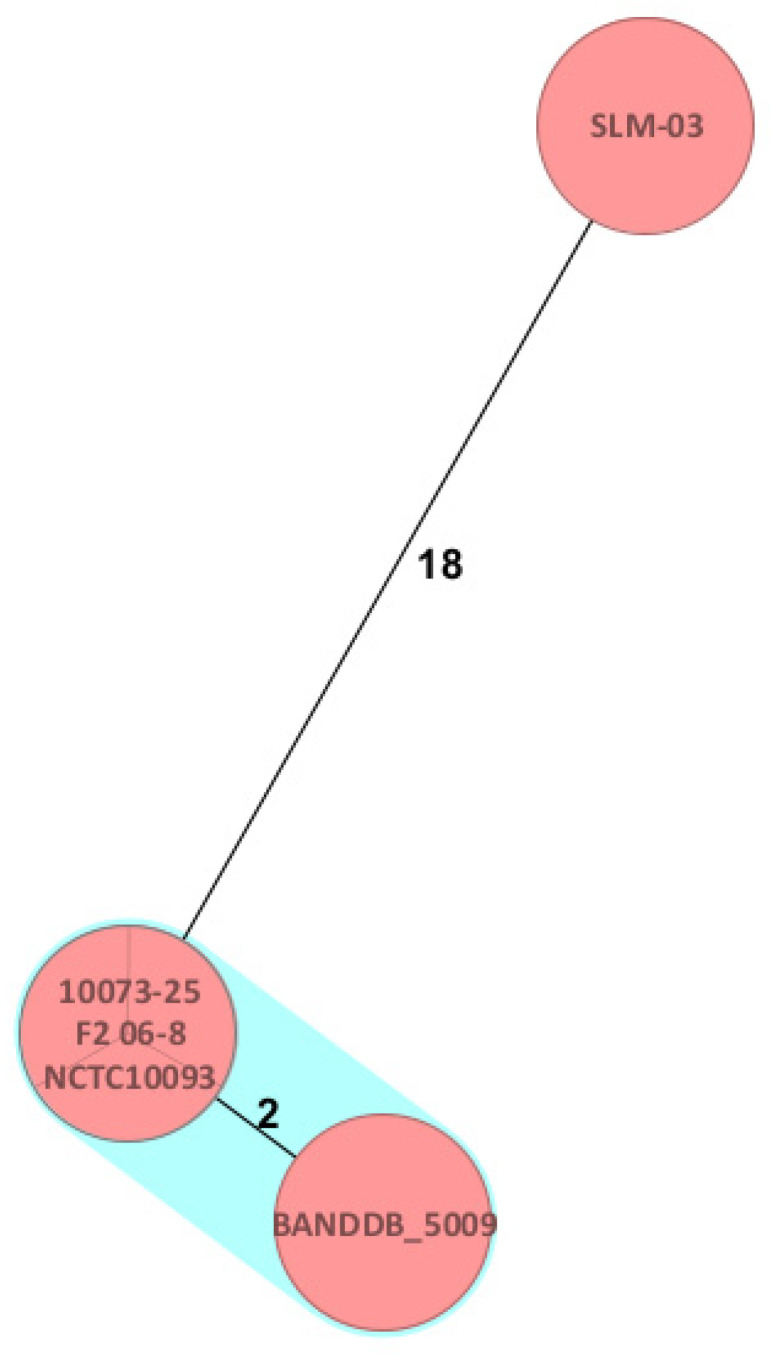
Minimum spanning tree generated using Ridom SeqSphere+ based on 1764 core genome multilocus sequence typing (cgMLST) alleles of the *Brucella abortus* biovar 1 isolate (10073-25) obtained from the present study. The cluster distance threshold was set to three allelic differences. Colors indicate sequence types (STs), with orange representing ST1. Identical isolates (NCTC10093 and F2 06-8) and the closely related isolate BANDDB_5009 are shown within the highlighted cluster. Numbers on the connecting lines indicate allelic distances. The full minimum spanning tree is provided in Supplementary Figure S1.

For whole genome sequencing (WGS), library preparation was performed using the Illumina DNA Prep Kit, (Illumina, San Diego, CA, USA) followed by paired-end sequencing (2 × 150 bp) on a NextSeq 550 (Illumina, San Diego, CA, USA) platform. De novo assembly and core genome multilocus sequence typing (cgMLST) analysis were performed using the *Brucella* scheme implemented in Ridom SeqSphere + (Ridom GmbH, Münster, Germany) version 10.5.3 software [21]. The isolate was compared with 375 publicly available *Brucella abortus* genomes retrieved from the National Center for Biotechnology Information (NCBI) database (Supplementary Table S1). Minimum spanning tree analysis revealed zero allelic differences between the study isolate 10073-2025, isolate (F2 06-8) and reference strain NCTC10093. BANDDB_5009 differed by two alleles, all clustering within complex type 285 (Figure 1). In silico multilocus sequence typing (MLST) assigned the isolate to sequence type 1 (ST1). 

Based on these findings, the laboratory confirmed the diagnosis of acute brucellosis. Following the initial diagnosis, a treatment regimen consisting of oral doxycycline (100 mg twice a day for 6 weeks) and rifampicin (600 mg once a day for 10 days) was initiated. After antibiotic treatment, the patient became asymptomatic; however, because of the possibility of relapse, long-term follow-up will continue for at least two years. The first 6-month check-up was completed, and EDTA blood was negative in qPCR; native blood was used for serology, and the Brucella antibody levels remained the same.

The epidemiological investigation suggested a possible laboratory-acquired infection (LAI). The patient was the only individual directly involved in handling the sample. Therefore, no additional laboratory personnel were considered exposed. A structured risk assessment was performed, taking into account the type and duration of exposure, the absence of high-risk laboratory procedures, and the use of standard laboratory precautions. Based on this assessment, the risk of transmission was considered low.

No additional symptomatic cases were identified. Household contacts were not systematically evaluated. However, given the extremely low likelihood of human-to-human transmission of brucellosis, the risk of secondary transmission was considered minimal. No secondary cases were reported. There was no relevant travel history or animal exposure.

In addition to the index patient, three laboratory workers in the hospital laboratory were assessed as having low-risk exposure. However, given their work in a clinical laboratory setting and the handling of blood culture samples that were initially reported as negative, they were invited to participate in serological follow-up at baseline and at 6 months.

This hypothesis was further supported by the results of whole genome sequencing (WGS). The patient worked in a diagnostic microbiology laboratory and had recent occupational exposure to bacterial cultures, including reference strains. Although no specific exposure event was identified, unrecognized aerosol exposure during routine laboratory procedures was considered the most plausible route of infection, consistent with previously reported laboratory-acquired brucellosis cases. The case will be included in the annual brucellosis surveillance report submitted to the European Centre for Disease Prevention and Control (ECDC).

## 3. Discussion

Laboratory-acquired brucellosis is one of the most frequently reported laboratory-associated bacterial infections. Transmission typically occurs through aerosolization of organisms during manipulation of cultures, centrifugation, or accidental exposure to infected specimens. Because *Brucella* species are highly infectious and can be transmitted by very low inoculum, biosafety level 3 (BSL-3) laboratory practices are recommended when handling suspected isolates [18].

In non-endemic settings, brucellosis is often not considered in the differential diagnosis of prolonged febrile illness. The presence of systemic inflammation and autoimmune markers, such as positive antinuclear antibodies, may further mislead clinicians toward autoimmune disease, potentially delaying appropriate antimicrobial treatment [13].

In our patient, the constellation of persistent undulant fever, arthralgia, elevated inflammatory markers (CRP 34 mg/L), elevated ferritin levels (146 ng/mL), and abnormal liver enzymes (ALT 147 U/L, AST 53 U/L) created a clinical picture suggestive of a systemic inflammatory or autoimmune disease. The detection of antinuclear antibodies (ANA) at a titer of 1:160 with a granular pattern further reinforced this suspicion. Although ANA positivity at this titer is not disease-specific, it is frequently interpreted as supportive evidence of connective tissue disease in the appropriate clinical context [1,13,22].

Brucellosis is a well-recognized infectious mimic of systemic autoimmune diseases. Beyond isolated case reports, several studies have described autoimmune-like manifestations, including autoantibody positivity and systemic inflammatory features, in patients with brucellosis [1,9,12,14,23,24]. Its nonspecific clinical presentation, together with laboratory abnormalities such as elevated acute-phase reactants and autoantibody positivity, may closely resemble systemic lupus erythematosus or other connective tissue disorders. Identifying and mitigating infectious risk factors is a critical component of clinical history. This requires a thorough assessment of the patient’s occupational environment, including direct exposure to animals and specific hazardous materials or environmental conditions. From a pathophysiological perspective, this case illustrates infection-driven immune activation rather than primary autoimmunity. The inflammatory profile and mild ferritin elevation may reflect underlying inflammatory processes associated with brucellosis, as elevated ferritin levels have also been described in previous reports [11,12]. Premature initiation of immunosuppressive therapy in unrecognized brucellosis may facilitate bacterial dissemination and delay appropriate antimicrobial treatment.

The negative initial blood culture further contributed to diagnostic uncertainty. The sensitivity of blood cultures in brucellosis depends on multiple factors, including disease stage, bacterial load, and sample volume, and false-negative results are not uncommon [13].

In this case, the diagnosis was ultimately established through repeat blood cultures and targeted molecular testing. The discordance between the negative serum qPCR and the positive EDTA blood qPCR highlights the importance of appropriate specimen selection in molecular diagnostics. Overall, this case emphasizes that the combined application of serological, microbiological, and molecular diagnostic methods is essential for accurate and timely diagnosis, particularly in low-incidence settings where clinical suspicion may be limited [13].

Genomic analysis identified the isolate as *Brucella abortus* sequence type 1 (ST1), a lineage that is widely distributed globally and historically associated with bovine reservoirs [25]. However, core genome multilocus sequence typing (cgMLST) revealed that the isolate was indistinguishable from the NCTC10093 reference strain, which is commonly used in laboratories [23]. This close genomic similarity strongly suggests a laboratory-acquired infection rather than exposure to a natural reservoir. These findings highlight the substantial added value of whole genome sequencing in accurately characterizing strains and supporting epidemiological interpretation, particularly in settings with low disease incidence.

*Brucella* species remain among the leading causes of laboratory-acquired infections (LAI), highlighting the importance of strict biosafety precautions during laboratory handling. Appropriate personal protective equipment, including laboratory coveralls, respirators, and protective gloves, is essential to minimize occupational exposure [1,2].

When brucellosis is suspected, obtaining blood cultures is crucial. Both native blood and EDTA blood specimens should be collected. In addition, samples from sites of clinical manifestation (e.g., cerebrospinal fluid, respiratory specimens, stool, or urine) may be useful depending on the clinical presentation. Although serological tests have limited sensitivity and specificity, they remain an important component of the diagnostic workup for brucellosis [13].

The antimicrobial regimen used in this case differed from standard guideline recommendations, which typically include a longer duration of rifampicin in combination therapy. Nevertheless, the patient achieved complete clinical recovery without evidence of relapse during follow-up, indicating a favorable outcome in this individual case.

It is important to note that serological markers may remain positive for prolonged periods following successful treatment of brucellosis. In our case, antibody levels remained detectable at the 6-month follow-up despite complete clinical recovery and negative PCR results. Therefore, clinical resolution in combination with negative molecular findings may represent a more reliable indicator of treatment success than serological normalization alone.

This case emphasizes the importance of clinical awareness of brucellosis and the potential for laboratory-acquired infection among microbiology personnel, even in regions where the disease is rare.

## 4. Conclusions

This case highlights acute brucellosis as a paradigmatic example of infection-induced immune dysregulation mimicking systemic autoimmune disease. The combination of undulant fever, arthralgia, elevated inflammatory markers, autoantibody positivity, and elevated ferritin levels created a clinical scenario strongly suggestive of autoimmune pathology and nearly led to the initiation of corticosteroid therapy. Timely microbiological and genomic confirmation prevented inappropriate immunosuppression, enabled targeted antimicrobial treatment, and contributed to elucidating the epidemiological background of the case. Maintaining a high index of suspicion for zoonotic infections—even in regions with low reported incidence—is essential to avoid diagnostic delay and to distinguish infection-driven inflammation from primary autoimmune disease.

## Data Availability

The sequence has been deposited at the GenBank under the accession of SUB15330836 BioSample: PRJNA1277139.

## Supplementary Materials

The following supporting information can be downloaded at https://www.mdpi.com/article/10.3390/pathogens15050460/s1, Table S1: *Brucella abortus* isolates used in this study to perform cgMLST. Figure S1: Full minimum spanning tree based on cgMLST analysis of *Brucella abortus* isolates. Due to the complexity of the dataset, the complete network is presented as Supplementary Material.

## Abbreviations

ANA	Antinuclear Antibody
ALT	Alanine Aminotransferase
AST	Aspartate Aminotransferase
BSL-3	Biosafety Level 3
cgMLST	Core Genome Multilocus Sequence Typing
CRP	C-reactive Protein
DNA	Deoxyribonucleic Acid
EDTA	Ethylenediaminetetraacetic Acid
ECDC	European Centre for Disease Prevention and Control
ELISA	Enzyme-Linked Immunosorbent Assay
EUCAST	European Committee on Antimicrobial Susceptibility Testing
GFAP	Glial Fibrillary Acidic Protein
IgA	Immunoglobulin A
IgG	Immunoglobulin G
IgM	Immunoglobulin M
LAI	Laboratory-Acquired Infection
MALDI-TOF MS	Matrix-Assisted Laser Desorption/Ionization Time-of-Flight Mass Spectrometry
MIC	Minimum Inhibitory Concentration
MLST	Multilocus Sequence Typing
NCBI	National Center for Biotechnology Information
PCR	Polymerase Chain Reaction
qPCR	Quantitative Polymerase Chain Reaction
ST	Sequence Type
WGS	Whole Genome Sequencing
