# Supplementary material for: Laboratory-Acquired Brucella abortus Infection Mimicking Autoimmune Disease: A Case Report with Genomic Confirmation
Source: Pathogens. 2026 Apr 23;15(5):460. doi: 10.3390/pathogens15050460 (PMC13209820; doi:10.3390/pathogens15050460)
Supplement: Supplementary file 1 [file pathogens-15-00460-s001.zip › pathogens-4249800-supplementary figS1.pdf]

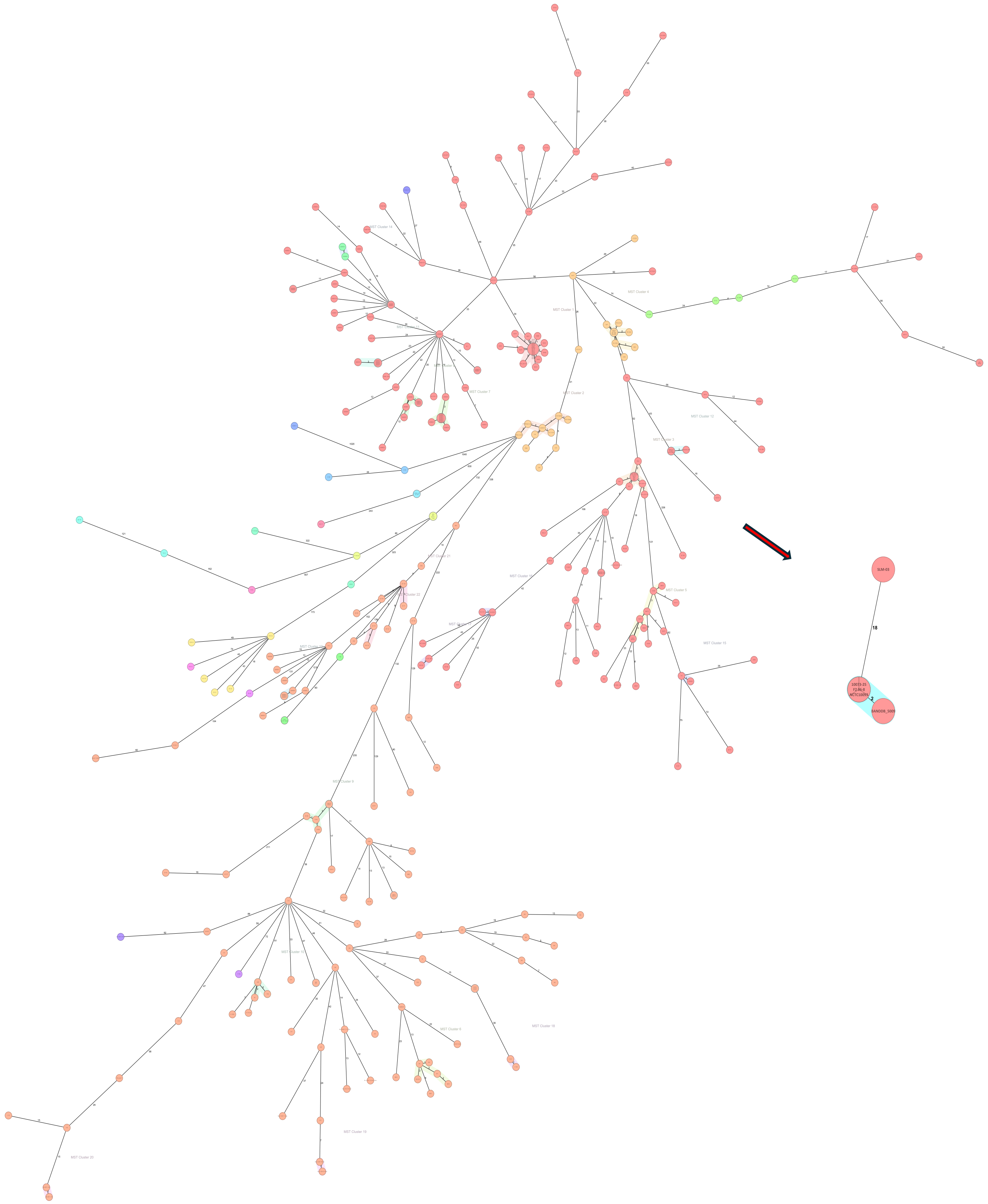

Figure S1. Full minimum spanning tree generated using Ridom SeqSphere+ based on 1764 core genome multilocus sequence typing (cgMLST) alleles of *Brucella abortus* isolates. The cluster

distance threshold was set to three allelic differences. Colors indicate sequence types (STs), with orange representing ST1. The red arrow indicates the highlighted area containing the study isolate and neighboring isolates with varying allelic distances. Numbers on the connecting lines represent allelic distances.
